# The flavor-enhancing action of glutamate and its mechanism involving the notion of kokumi

**DOI:** 10.1038/s41538-023-00178-2

**Published:** 2023-01-27

**Authors:** Takashi Yamamoto, Chizuko Inui-Yamamoto

**Affiliations:** 1 Health Science Research Center, Kio University, 4-2-4 Umami-naka, Koryo, Kitakatsuragi, Nara, 635-0832 Japan; 2 Department of Oral Anatomy and Developmental Biology, Osaka University Graduate School of Dentistry, 1-8 Yamadaoka, Suita, Osaka, 565-0871 Japan

**Keywords:** Taste receptors, Feeding behaviour

## Abstract

The sodium salt of glutamic acid, or monosodium glutamate (MSG), has two effects in foods: one is to induce a unique taste called umami, which is one of the five basic tastes, and the other is to make food palatable (i.e., flavor-enhancing or seasoning effects). However, the mechanism behind how MSG makes food more palatable remains poorly understood, although many food scientists seem to believe that the umami taste itself plays an important role. Here, we propose an alternative notion regarding this topic based on previous and recent studies. When added to complex food compositions, MSG facilitates the binding of existing *kokumi* substances to *kokumi* receptors. In turn, these bound *kokumi* substances enhance the intensity of umami, sweet, salty, and fatty tastes, resulting in increased palatability accompanied by *kokumi* flavor, such as thickness, mouthfulness, and continuity. The requisite for sufficient palatability and *kokumi* flavor is a good balance of umami and *kokumi* substances. This framework gives a scientifically useful background for providing newly developed foods, including cultured meat and plant-based meat substitutes, with good taste characteristics.

## Introduction

One of the main purposes of eating is to enjoy the palatability of foods. The eating of palatable foods is good for mental and physical health if overeating can be avoided^1–4^. There are two types of palatability of foods: one comes from the savory primary constituents of meals, and the other from desserts and confectioneries. As the purpose of the primary components of meals is to supply nutrients and calories from its ingredients, such foods should be palatable to increase the pleasure derived from their consumption. Palatability evaluations may differ among people around the world because palatability is often related to individuals’ early experiences in the local food culture in addition to innate preferences^5–8^. Different kinds of palatable cuisine around the world are commonly known to contain a high amount of umami-related substances, namely glutamate, inosinate, and guanylate, regardless of ethnic, regional, and historical differences^9,10^, which suggests that umami substances play an important role in making food palatable. However, the precise role of these substances has not yet been well elucidated. On the other hand, the palatability of desserts and confectioneries, such as cakes, sweets, and ice cream, is mainly based on high amounts of sugars and fats with the supplementation of a low concentration of NaCl; these substances are innately preferred regardless of ethnic, sex, or age differences.

To our knowledge, no studies have been conducted reexamining why umami substances make food palatable, although many food scientists seem to believe that the umami taste itself plays an important role (e.g.,^11,12^). Therefore, the present article aimed to focus on the palatability of foods and elucidate the mechanisms underlying the function of umami substances in making processed foods more palatable. The results can be expected to provide hints for offering improved taste to food products that are rapidly growing in popularity, such as cultured meat and plant-based burger patties made from potatoes, rice, and peas.

## Proposal of umami as being distinct from the four basic tastes

The discovery of umami substances was made by a Japanese scientist, Kikunae Ikeda, more than 100 years ago^13^. During his stay in Leipzig, Germany, to study chemistry with Prof. W. Ostwald, Ikeda recognized a peculiar taste in such foods as tomatoes, cheese, asparagus, and meat, which he thought to be different from the conventional four basic tastes of sweet, salty, sour, and bitter. After returning to Japan, he noticed the same peculiar taste in cuisine flavored with *kombu* (kelp, seaweed). Before identifying the key substance that induced this unique taste, he tentatively called it *umami* (literally, “palatable taste”) because it is closely related to the palatability of foods such as meat, fish, and soup stocks made from *kombu* or dried bonito^13^.

## Discovery of the essence of umami

Subsequently, Ikeda attempted to identify a compound that could induce umami. As little as 6 g of a crystalline substance, which he identified as L-glutamic acid, was successfully obtained from 30 kg of kombu^13^. When he tasted the crystal, he immediately recognized that the taste was the same as that he had been looking for— the essence of umami. He obtained a patent for the manufacture of monosodium glutamate (MSG) which involves replacing one of the H ions in L-glutamic acid with an Na ion; this compound induces pure umami without a sour taste.

After the discovery of MSG, inosinate (5’-inosine monophosphate [IMP], a 5’-ribonucleotide) was found to be the key element in the palatability of *katsuobushi* (dried bonito), which also elicited umami^14^. Guanylate (5’-guanylate monophosphate [GMP], a 5’-ribonucleotide) was also found to have umami^15^, and this substance was later found to exist abundantly in mushrooms. The salts of L-glutamate, 5’-inosinate, and 5’-guanylate are globally used in the market and are collectively called umami substances.

The taste synergism between glutamate and 5’-ribonucleotides (inosinate, guanylate, and adenylate) is a hallmark of umami, and the intensity of umami is markedly enhanced when both types of umami substances are mixed^15–19^. The mechanisms of umami potentiation have now been elucidated at the receptor level^20^.

## Characteristics of umami

In 1985, an international symposium on umami was held in Hawaii after a long silence following Ikeda’s first report in 1909. Among various presentations from different fields, Yamaguchi^21^ showed in a human psychophysical study that umami is the fifth basic taste and could not be explained by the four conventional basic tastes using multidimensional analysis. Although some opinions were expressed against the position of umami as a basic taste, the decisive evidence came with the discovery of receptors for umami. It is now accepted that there are at least two types of umami receptors: T1R1/T1R3 heterodimer^22,23^, and metabotropic glutamate receptors (mGluR1 and mGluR4)^24,25^ on the taste cell membrane.

How can the properties of umami (or umami taste) be described? Umami is a delicate and subtle taste buried within other tastes in foods, and thus, only discerning persons, such as Ikeda, can identify it in natural ingredients.

If you taste a small amount of pure MSG, you can easily recognize the taste and discriminate it from others. Some sensory tests have been conducted to elucidate the characteristics of umami (For a review, see ^26^). The results were as follows: 1)The slope of the concentration–intensity function of MSG, which follows Fechner’s law, is not particularly steep compared with that for other basic tastes, and the strongest umami is weaker than the strongest taste elicited by other basic taste stimuli, indicating that umami does not become extremely strong even at high concentrations of MSG; 2) When small areas of the tongue were separately stimulated with various umami stimuli using a filter paper method, umami was most strongly sensed when the foliate papillae, which are located on the posterior part of the tongue, were stimulated, indicating that a large number of umami receptors are simultaneously stimulated at the timing of swallowing leading to an aftertaste of umami; 3) The participants were asked to lick a small amount (0.01 mL) of taste solution on a spoon and to indicate on which part of the tongue they could perceive the taste of the solution. Although each of the basic taste stimuli stimulated the anterior part of the tongue, only umami was sensed over a wide area of the tongue, including the middle of the tongue, where there are no taste buds, indicating that the area in which taste was perceived did not always reflect the true sensitive loci when a small amount of solution was sampled^27,28^. Such a disparity may be explained in part by illusion or phantom sensation^29,30^. This type of taste sensation might be related to the spatial effects of the umami taste such as broad development and mouthfulness;^26–28^ and 4) Umami lingers longer than other tastes after swallowing^31^.

As described above, umami is not easily recognized in natural ingredients in comparison with other basic tastes. Moreover, accumulating data suggest that MSG plays important roles in the gastrointestinal tract after swallowing^32–35^. Therefore, some researchers define umami as an alimentary rather than an oral taste^36^. However, it seems clear that umami is a unique taste and can be discriminated from other tastes in the oral cavity, even though some people cannot recognize umami or discriminate MSG from NaCl^37^ owing to their limited experience with MSG^9,38^ or genetic issues involving umami receptors^39^.

## Is umami palatable?

Is the unique and subtle taste of umami preferable or hedonically positive? In infants, MSG dissolved in water is rejected dose-dependently^40,41^. In adults, aqueous solutions of MSG are not preferred in any tested concentrations^42^, essentially no effects are observed when MSG is added to other basic tastes, and other tastes do not influence the hedonics of umami^43^. These findings suggest that umami itself, especially when tasted in a water solution, is not innately preferred by humans.

This seems to be contradictory to the fact that umami-rich foods are typically considered to be delicious as well as to Ikeda’s naming of umami based on his experience that “umami is a palatable taste”. Two possible explanations may be available for this discrepancy: one is that the hedonics of umami are different in food (or complex compositions of ingredients) compared with those in water and become highly positive, but no supporting evidence is available for this idea. The other explanation is that of a conditioned taste preference, which refers to taste preference learning (i.e., when the ingestion of novel food is associated with preferable oral and/or post-oral consequences) the food becomes preferred on the basis of its taste as a cue^44^. Food becomes palatable when it contains MSG, as discussed in the following sections, and umami, if perceived, also becomes preferred and acceptable after repeated intake of MSG-containing palatable food.

## Additive effects of glutamate

Another important and fascinating function of MSG is its flavor-enhancing action: its addition to prepared foods can improve both palatability and satisfaction^3,45,46^. A well-known phenomenon is that human infants exhibit positive facial expressions indicating preferability when MSG is added to vegetable soup^40,47^, suggesting that the mixture of MSG and vegetable soup becomes more palatable than vegetable soup alone. It is also well accepted that low-NaCl foods, which are recommended to avoid excessive sodium intake, but are not sufficiently satisfactory to consumers, become more palatable and satisfactory after supplementation with MSG^28,48–52^. To improve food intake in older persons and hospitalized patients, the addition of MSG has been reported to be very effective in enhancing both appetite and consumption^53,54^.

What happens when MSG is added to foods? According to comments from famous chefs, umami substances increase the flavor of the ingredients of foods^55^. For example, “Discovering umami gives us a chance to create dishes that are irresistible even with just a few ingredients, because it brings the natural deliciousness of those ingredients to the fore” (Michael Anthony, Chef, Gramercy Tavern, USA), and “Umami creates deep taste and harmony. By combining umami ingredients, I can come up with dishes that are balanced and full of rich flavor” (Pedro Miguel Schiaffino, Owner chef, Malabar, Peru). More scientifically, the addition of MSG to beef consommé has been shown to enhance overall taste intensity and increase flavor characteristics such as thickness, continuity, mouthfulness, impact, and roundedness, leading to its increased overall palatability and preferability^26,56^. All these terms are closely related with each other and form the concept of “*koku*” or “*kokumi*”. Japanese people generally use the conceptual word, *koku*, on a daily basis when they evaluate the palatability of foods^57^. *Kokumi*, on the other hand, is a coined term by Ueda et al.^58^ to denote essentially the same concept as *koku* which is induced in a simple experimental situation where the taste of an umami solution is modified by adding a certain substance. As *kokumi* rather than *koku* is now widely used in scientific papers in the fields of taste physiology and food sciences, *kokumi* is used in this article instead of *koku* hereafter.

It should be noted here again that aqueous solutions of MSG are not palatable, and that MSG does not affect the palatability of other basic tastes. MSG exerts its palatability-enhancing actions only when added to complex food ingredients. In the consommé experiment, the concentration of MSG used was as low as 0.05%, which is near the sensory threshold of MSG and gives no clear umami taste. Therefore, the role of umami substances in making food palatable may not be due to a simple addition of umami itself, but rather, to different mechanisms involving increase of *kokumi* associated with increased palatability, which will be described precisely later in this article.

## What is *kokumi*?

As suggested above, an important key word with regard to the flavor-enhancing action of MSG is *kokumi*, which literally means strong, rich, or concentrated, and is usually associated with increased palatability. *Kokumi* is related to the quantitative aspect of sensations and positive hedonics. Generally, *kokumi* (or *kokumi* flavor) is induced predominantly by combinations of different senses, such as the smell, texture, and taste of food containing complex compounds, which are generally obtained after maturation, fermentation, aging, curing, drying, or slow cooking^57^.

To evaluate *kokumi* scientifically in sensory tests, the attributes of *kokumi* are used, such as thickness (concentration, amplitude, strength, but not viscosity), mouthfulness (the spread of sensation throughout the whole mouth), continuity (long-lasting sensory effects including an increase in duration of aftertaste), roundedness (smoothness, balance, harmony), depth (richness, complexity), and punch (impact, quick increase). Some of these terms are translated from Japanese terms, leading to some confusions in their interpretations. The language issues should be addressed elsewhere.

**Fig. 1:**
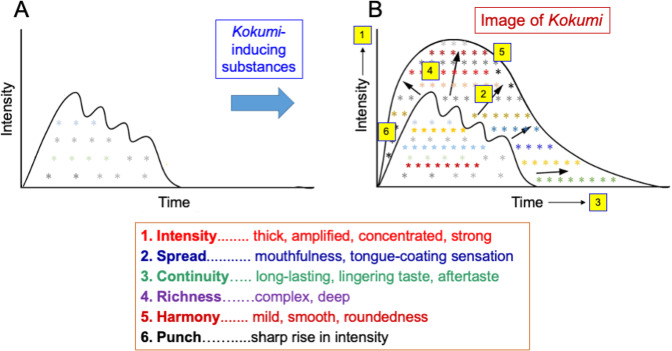
A theoretical representation of kokumi with six related characteristics. Under the influence of *kokumi* substances, food with umami and other taste substances (**A**) becomes more palatable with the *kokumi* attributes (**B**). This image corresponds to stages C and D in Fig. 2.

These attributes can be explained by the visual representation of *kokumi* shown in Fig. 1, where thickness is represented by the increase of ordinate, continuity by the increase of abscissa, mouthfulness, by the increase of the area, roundedness by the uneven to smooth curve, depth by the small to large numbers of symbols, and punch by the steep increase of the onset of the curve.

On the other hand, Ueda et al.^58^ showed that a certain single chemical substance without odor or texture elicited *kokumi* flavor when added to umami solutions. They used a relatively simple model consisting of odorless garlic extract as a substance and a mixture of 0.05% MSG and 0.05% IMP as an aqueous umami solution. They named the substance a “*kokumi* substance” and the induced effect *kokumi* (or *kokumi* flavor); it gave umami such characteristics as thickness, mouthfulness, and continuity corresponding to increased intensity, an enhanced spreading sensation, and a greater duration of lingering of the umami taste characteristics. However, this aqueous umami solution was not assessed to be palatable. In subsequent studies, Ueda et al. used sulfur-containing components in onion^59^ and glutathione^60^ as *kokumi* substances and showed that these substances increased the *kokumi* flavor of the umami solution. Following these findings, Dunkel et al.^61^ suggested that γ-glutamyl peptides isolated from beans are also *kokumi* flavor compounds, and Toelstede et al.^62^ reported that a series of *kokumi* peptides imparted the long-lasting mouthfulness of aged Gouda cheese.

In 2010, Ohsu et al.^63^ published an important paper on a possible receptor for *kokumi* substances. They found that among 46 γ-glutamyl peptides, glutathione (γ-glutamyl-cysteinyl-glycine) and γ-glutamyl-valinyl-glycine (γ-Glu-Val-Gly) were very strong calcium-sensing receptor (CaSR) agonists that increased the *kokumi* flavor of chicken consommé and enhanced sweet, salty, and umami tastes independently when added to aqueous solutions of sucrose, NaCl, and MSG, respectively, in human sensory tests. These *kokumi* substances bind CaSR expressed in taste tissue^64^ to exert their effects at concentrations where they induce no taste of their own, which is a characteristic of *kokumi* substances^63^. Maruyama et al.^65^ demonstrated that γ-Glu-Val-Gly activated a subset of taste-responsive type II and type III cells in taste buds. These findings suggest that information from cells in which CaSR agonists have bound to the receptors might not be sent to the brain, but may instead be used to modify other taste responses within taste buds. The CaSR existing in the parathyroid gland and kidney is known to be critical for the maintenance of blood calcium in a narrow physiological range^66^. The CaSR expressed in the taste bud cells may be a variant of in vivo CaSR because calcium concentrations in the oral cavity are highly variable. The type of CaSR involved in *kokumi* flavor should be further elucidated in future studies.

As such, *kokumi* flavor, *kokumi* substances, and *kokumi* receptors are closely linked. Some researchers assume to take *kokumi* as a taste quality^36,67^, but it is noted here again that *kokumi* denotes a quantitative aspect of flavor, such as intensity, spread, or continuity.

## Function of *kokumi* substances

To elucidate the basic function of *kokumi* substances, behavioral and electrophysiological experiments were performed using glutathione and γ-Glu-Val-Gly in rodents. Yamamoto et al.^68^ conducted experiments to explore the combined effects of glutathione and umami substances in mice, and found that the addition of glutathione to IMP, rather than monopotassium glutamate, increased preference for the umami solution. Neural responses of the taste nerves showed synergism with the mixture of glutathione and IMP. They suggested that glutathione increased the preference for 5’-ribonucletides (IMP) more than glutamate. Yamamoto and Mizuta^69^ reported that at low concentrations that do not elicit a taste of its own, γ-Glu-Val-Gly increases preferences for umami, fatty, and sweet taste solutions in rats. An increased preference for IMP and a soybean oil emulsion was the most dominant effect. NPS-2143, a CaSR antagonist, abolished the additive effect of γ-Glu-Val-Gly on the IMP and soybean oil emulsion solutions. These effects were electrophysiologically verified by taste nerve responses.

More detailed information was obtained by Mizuta et al.^70^, who showed that ornithine (L-ornithine but not D-ornithine) at low concentrations increased preferences for sweet, salty, umami, and fatty taste solutions in mice. In contrast to glutathione and γ-Glu-Val-Gly, ornithine increased the preference for MSG more dominantly than IMP. Antagonists of G-protein-coupled receptor family C group 6 subtype A (GPRC6A) abolished the additive effect of ornithine on preference for MSG solutions in both behavioral and electrophysiological experiments, suggesting that ornithine is a *kokumi* substance that binds to another possible *kokumi* receptor, GPRC6A.

These in vivo animal experiments suggest that *kokumi* substances can increase preferences for sweet, salty, umami, and fatty substances, and especially for umami substances. These results may partially explain the underlying mechanisms that induce human perception of *kokumi* flavor associated with palatability. Although we should be careful in applying results from animals to humans because of the species differences, including the well-known differences in the sensitivity of umami receptors to amino acids^22,23^, some basic mechanisms may be common among species for searching and accepting more nutritional and palatable edibles.

In humans, when added to basic taste aqueous solutions, MSG does not enhance or suppress any tastes^71^ because no *kokumi* substances exist in these solutions. When MSG is added to complex food ingredients, the tastes of the ingredients are enhanced by the action of the existing *kokumi* substances. This notion is partly supported by an experiment showing that a vegetable soup highly preferred by rats became less preferred after the addition of CaSR antagonist, indicating that the strong preference was partly due to the action of *kokumi* substances binding to CaSR (Supplementary Fig. 1). Although some *kokumi* substances and *kokumi* receptors have been well studied, as described above, there are number of unknown or unidentified potential *kokumi* substances, including free amino acids, oligopeptides, vitamins, and minerals. For example, the addition of vitamin B_3_ (nicotinic acid and nicotinamide) increased the preference for MSG in rats (Supplementary Fig. 2); however, whether the effects of this vitamin involve any *kokumi* receptors remains unknown. Concerning this point, a recent study revealed that methional, a familiar flavor compound found in foods such as tomatoes and cheese, can significantly enhance responses to MSG by interacting with the transmembrane domain of T1R1 of the human umami receptor (T1R1/T1R3)^72^. This finding is quite interesting because methional directly interacts with the umami receptor and allosterically enhances MSG responses without involving the *kokumi* receptors. These findings suggest that *kokumi* substances are divided into at least two groups: one that exerts its effect via interactions with *kokumi* receptors, and another that directly binds with T1Rs and other G-protein-coupled receptors and exerts its effect by allosteric modulation of these receptors.

Leijon et al.^73^ recently reported that the oral application of *kokumi* substances including γ-Glu-Val-Gly elicited small responses with variable latencies ranging from 2 to over 200 s in a very small fraction (0.6%) of trigeminal neurons in mice. Co-application of a CaSR antagonist decreased these responses, indicating the involvement of CaSR. These findings offer suggestive evidence for the involvement of CaSR in trigeminal neuron responses to *kokumi* substances. However, further studies are needed to elucidate how significantly oral texture perceptions contribute to *kokumi* flavor.

## Interactions between *kokumi* and umami substances

As already described, the addition of MSG to ingredients while cooking increases the *kokumi* flavor and palatability of the food. *Kokumi* is a conceptual word denoting the quantitative aspects of flavor, such as thickness, mouthfulness, and continuity, which are independent of taste quality. However, why *kokumi* flavor is increased in association with palatability by the addition of MSG in complex compounds, but not in basic taste aqueous solutions, remains poorly understood. Animal experiments^68–70^ and a human psychophysical study^63^ suggest that these *kokumi* attributes come essentially from the enhanced action of MSG, while increased palatability comes from enhancement of sweet, salty, and fatty tastes, as well as umami, which are all related to food palatability, via the action of *kokumi* substances. This may explain the occurrence of palatability enhancement with *kokumi*. However, it remains a mystery why the addition of only MSG increases the overall palatability of food rather than simply increasing umami.

**Fig. 2:**
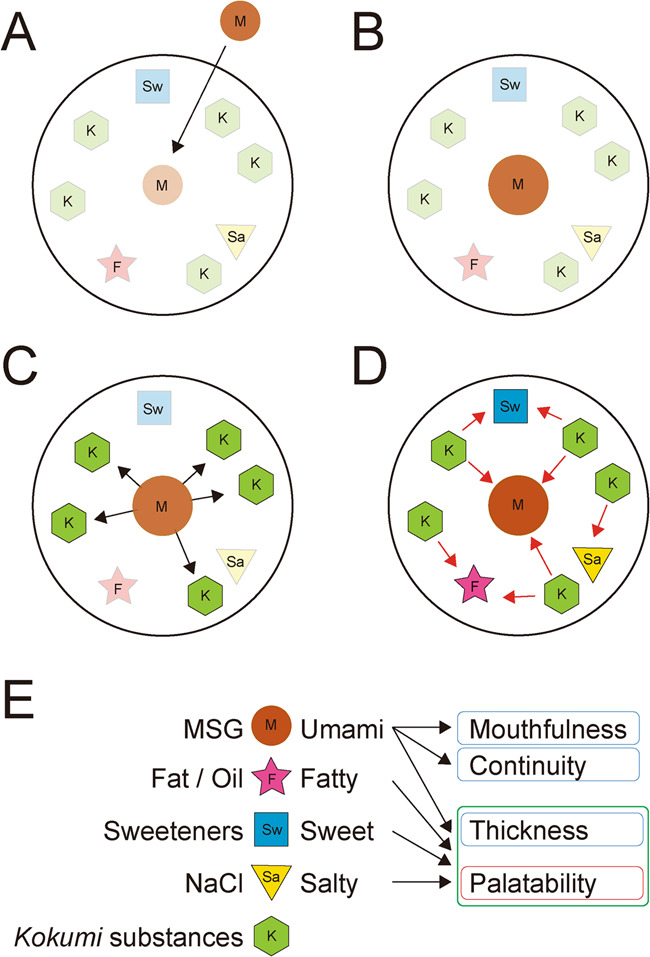
A theoretical representation of the mechanism underlying how the addition of monosodium glutamate (MSG) induces kokumi and palatability in food containing kokumi and basic taste substances. When MSG is added to food ingredients (**A**), the amount of MSG is increased (**B**). Increased MSG facilitates the binding of kokumi substances to kokumi receptors (**C**). The bound kokumi substances facilitate the binding of sweet, salty, and fatty substances to their corresponding receptors and enhances those tastes (**D**). Enhanced umami induces mouthfulness and continuity of sensation. Thickness comes from enhanced perception of the four tastes, and palatability is also induced on the basis of these palatable tastes (**E**).

As schematically illustrated in Fig. 2, one explanation for this phenomenon is that the presence of MSG facilitates the binding of *kokumi* substances to the kokumi receptors with higher affinity, as suggested by Ueda et al.^60^ and Dunkel et al.^61^ in the case of glutathione and γ-glutamyl peptides, respectively. There is a possibility that different kokumi substances, such as γ-glutamyl peptides and oligosaccharides, synergistically activate CaSR^74^. In turn, bound *kokumi* substances enhance sweet, salty, and fatty tastes, as well as umami, leading to an overall increase in *kokumi* and palatability. Unfortunately, except for the function of methional, direct interactions between MSG (or umami substances) and *kokumi* substances as well as *kokumi* substances and other basic taste receptors remain poorly elucidated at the receptor and cellular levels in taste buds. These crucial interactions await further research.

In summary, the characteristics of *kokumi*, such as mouthfulness and continuity (i.e., MSG’s spreading sensation on the tongue and lingering sensation, respectively), may come mainly from the properties of MSG (and/or other umami substances). In addition, the sensation of thickness may come from the simultaneous enhancement of sweet, salty, and fatty tastes, as well as umami, and eventually accompany palatability.

## Utilization of *kokumi* and umami substances to make food palatable

It is necessary to maintain a good balance between *kokumi* and umami substances to obtain sufficient *kokumi* flavor with palatability in food. If the amount of *kokumi* substances is low, the excess supplementation of umami substances will not work well, and vice versa. In cases where the ingredients on their own are judged not to be sufficient, both *kokumi*- and umami-rich foodstuffs should be cooked together. Such foodstuffs include tomatoes, cheese, milk, eggs, and various fermented products. Recently, the market for plant-based meat substitutes made from ingredients such as rice, potatoes, and peas has been growing rapidly in a move reflecting more widespread environmental awareness. To provide these foods with good taste characteristics, the important roles of *kokumi* and umami substances should be kept in mind.

## Conclusion

When MSG and/or other umami substances are added to complex food ingredients, MSG activates any existing *kokumi* substances, and in turn, these activated *kokumi* substances activate MSG to induce mouthfulness and continuity. In addition to umami substances, those inducing sweet, salty, and fatty tastes, which already exist in the foodstuffs, are also activated to make foods richer, thicker, more complex, and more palatable. The combination and balance of MSG and *kokumi* substances is important for effectively increasing the *kokumi* flavor and palatability of food.

## Supplementary information

Supplementary Information

nr-reporting-summary

## Data Availability

Data sharing is not applicable to the main text. Data in the supplementary figures are available on reasonable request from the corresponding author.
