# Supplementary material for: The flavor-enhancing action of glutamate and its mechanism involving the notion of kokumi
Source: NPJ Sci Food. 2023 Jan 27;7:3. doi: 10.1038/s41538-023-00178-2 (PMC9883458; doi:10.1038/s41538-023-00178-2)
Supplement: Supplementary file 1 — Supplementary Information [file 41538_2023_178_MOESM1_ESM.pdf]

Supplementary Figure 1

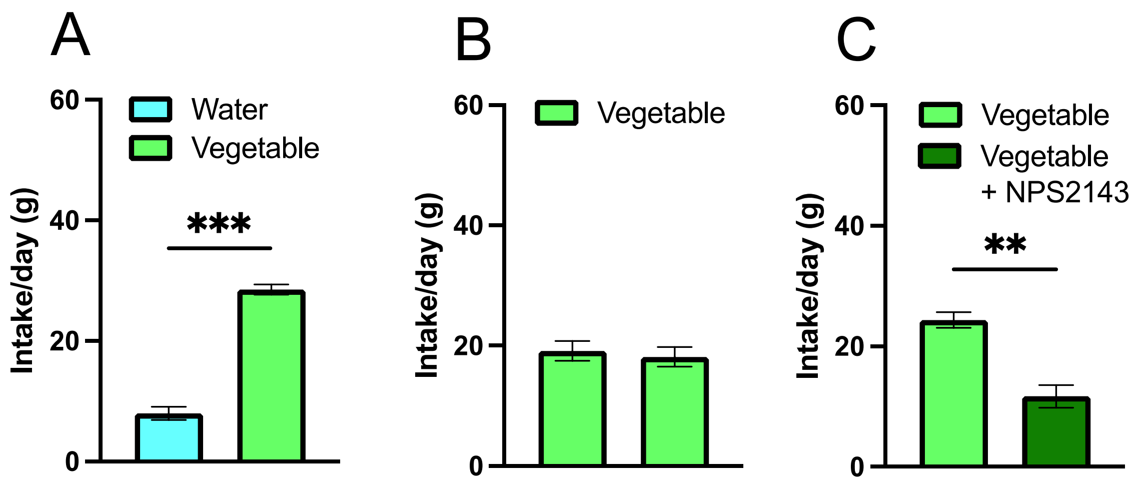

Effects of the calcium-sensing receptor (CaSR) antagonist, NPS-2143, on the preference for a vegetable soup in rats. Rats showed a stronger preference for vegetable soup than for plain water in a two-bottle preference test (A). When the same vegetable soup was presented in two bottles, almost the same amount was consumed from each bottle (B). When 0.001% NPS-2143 was added to either bottle containing the vegetable soup, rats showed a stronger preference for the vegetable soup without than that with the antagonist, indicating that preferences for vegetable soup involve CaSR receptors. The vegetable soup was prepared by dissolving a freeze-dried powder mixture of six different vegetables, a product of the Umami Information Center (Tokyo, Japan). NPS-2143 (Chemscene, Monmouth Junction, NJ, USA) was dissolved at a concentration of 0.001%, which was verified to have no taste for rats. Each value is mean ± SE; n = 7. **P<0.01, ***P<0.001 (paired *t*-test, two-tailed)

Supplementary Figure 2

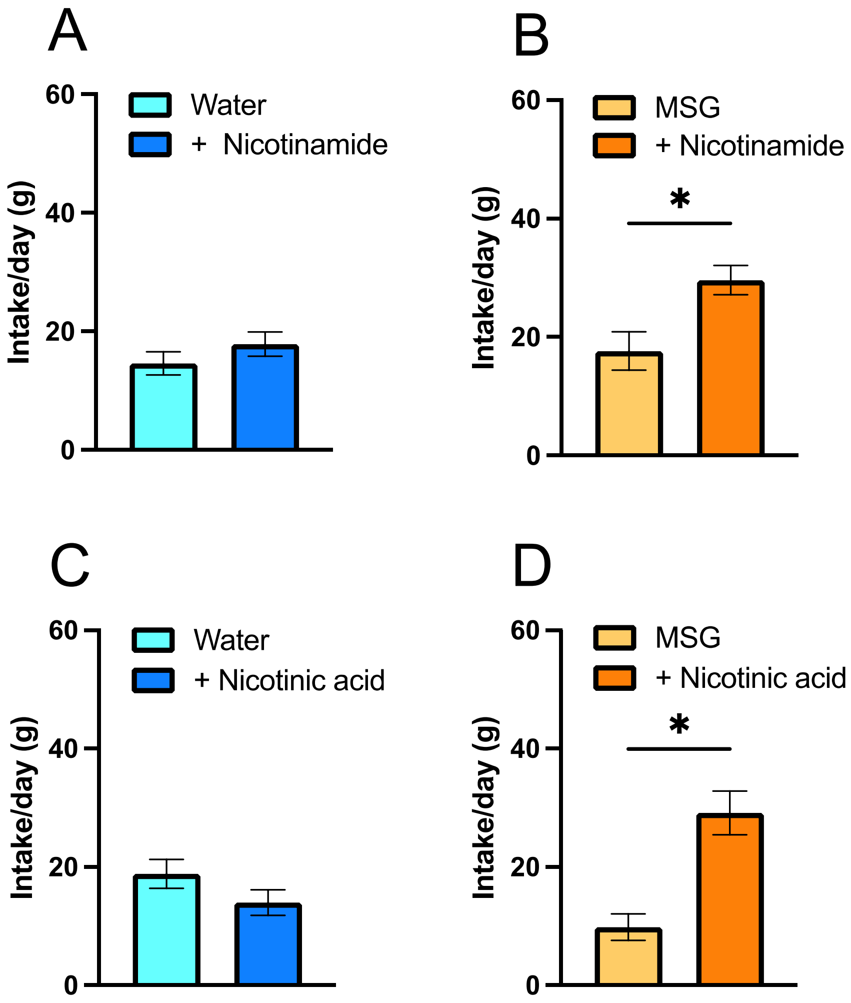

Effects of the supplementation of vitamin B_3 (nicotinamide and nicotinic acid) on water and monosodium glutamate (MSG) in a two-bottle preference test in rats. The addition of 0.003 M nicotinamide or 0.01 M nicotinic acid to water had no particular effect (A and C, respectively). The addition of these chemicals to 0.05 M MSG significantly increased the preference for it (B and D). These response characteristics are very similar to those observed for the *kokumi* substances γ-Glu-Val-Gly (69) and L-ornithine (70). Nicotinamide and nicotinic acid were purchased from Kanto Chemical (Tokyo, Japan). Each value is mean ± SE; n = 7. *P<0.05 (paired *t*-test, two-tailed)

Methods for animal experiments

Animals

Male Wistar rats were obtained from Japan SLC, Inc. (Shizuoka, Japan) at 8 weeks of age. The rats were housed in individual home cages in a temperature (25°C)- and humidity (60%)-controlled room on a 12:12 h light/dark cycle with lights on at 6:00 am. The tests were conducted during the light cycle. Animals had free access to food (CLEA Rodent Diet CE-2, CLEA Japan, Inc., Tokyo, Japan) and tap water. All animal care and experimental procedures conformed to the guidelines established by the National Institutes of Health, and the experimental protocols were approved by the Institutional Animal Care and Use Committee at Kio University (No. H30-10).

Behavioral experiment: two-bottle preference tests

Fourteen rats were randomly divided into 2 groups (7 of each): one group for "vegetable" experiment and the other for "Vitamin B3" experiment. Each animal was trained to drink distilled water from a stainless steel spout connected to a plastic bottle. The preference test was performed after a 1-week training period. The two-bottle preference test involved simultaneously presenting two bottles to each cage with stainless steel spouts. Each spout contained a ball inside to minimize the dripping of the liquid and had an inner diameter of 6 mm. Two spouts were separated by 5 cm from the center of each spout. The stimuli contained in the bottles are shown in each Supplementary Figure. The positions of the two bottles were switched at 24 h of the 48-h test session to account for any potential positional preference. The bottles with fluid were weighed before and after testing to measure the intake volume. The measurement was performed by a person who did not know the contents of the bottles. The total intake volume over 48 h was divided by 2 to obtain the intake volume per day.
